# Supplementary material for: Systems-based identification of the Hippo pathway for promoting fibrotic mesenchymal differentiation in systemic sclerosis
Source: Nat Commun. 2024 Jan 3;15:210. doi: 10.1038/s41467-023-44645-6 (PMC10764940; doi:10.1038/s41467-023-44645-6)
Supplement: Supplementary file 1 — Supplementary Information [file 41467_2023_44645_MOESM1_ESM.pdf]

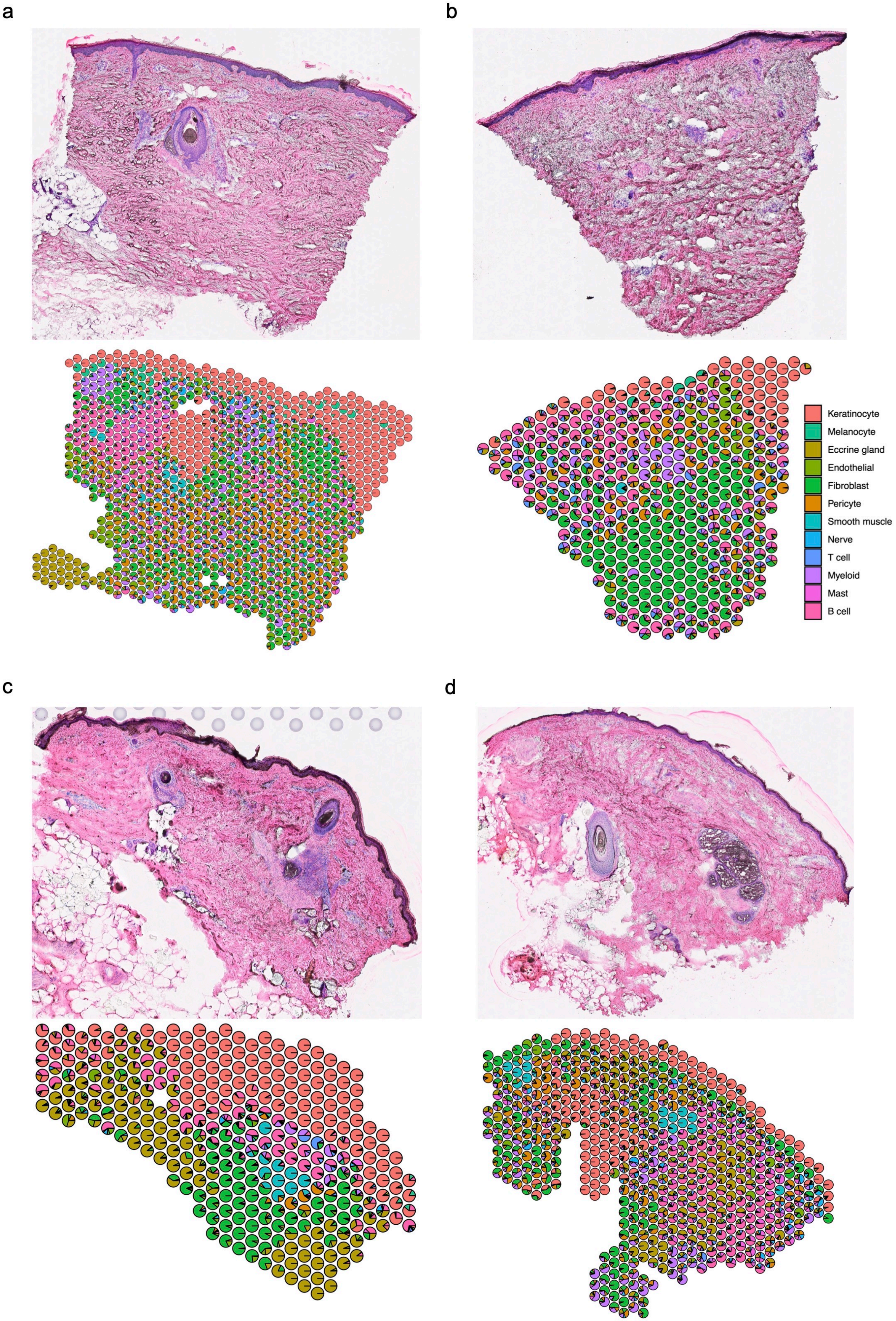
a
b
c
d
Keratinocyte
Melanocyte
Eccrine gland
Endothelial
Fibroblast
Pericyte
Smooth muscle
Nerve
T cell
Myeloid
Mast
B cell

Supplementary Fig. 1. Cell type deconvolution for four SSc spatial-seq samples.

a-d. Each panel contains one SSc spatial-seq sample. The top plot shows the H & E staining of the skin biopsy. The bottom scatter pie plot shows the cell type composition for each spot in the spatial-seq sample. Each spot is represented as a pie chart showing the relative proportion of the cell types.

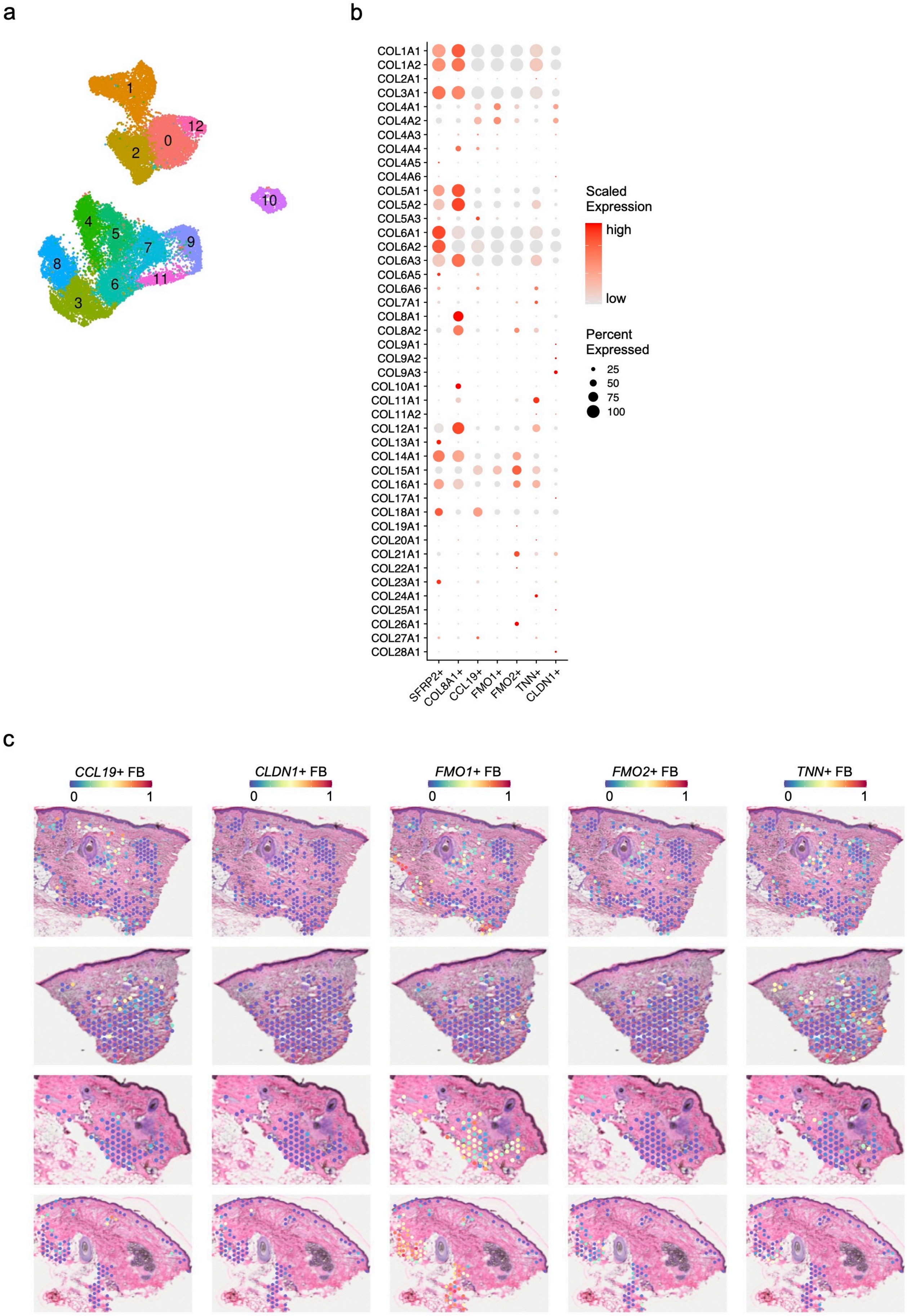

a
1
12
0
2
10
4
5
7
9
8
6
11
3
b
COL1A1
COL1A2
COL2A1
COL3A1
COL4A1
COL4A2
COL4A3
COL4A4
COL4A5
COL4A6
COL5A1
COL5A2
COL5A3
COL6A1
COL6A2
COL6A3
COL6A5
COL6A6
COL7A1
COL8A1
COL8A2
COL9A1
COL9A2
COL9A3
COL10A1
COL11A1
COL11A2
COL12A1
COL13A1
COL14A1
COL15A1
COL16A1
COL17A1
COL18A1
COL19A1
COL20A1
COL21A1
COL22A1
COL23A1
COL24A1
COL25A1
COL26A1
COL27A1
COL28A1
SFRP2+
COL8A1+
CCL19+
FMO1+
FMO2+
TNN+
CLDN1+
Scaled Expression
high
low
Percent Expressed
25
50
75
100
c
CCL19+ FB
CLDN1+ FB
FMO1+ FB
FMO2+ FB
TNN+ FB
0
1

Supplementary Fig. 2. Cell type deconvolution for four SSc spatial-seq samples.

a. UMAP plot showing 25,182 fibroblasts colored by sub-clusters.

b. Dot plot showing the expression of all the collagen genes across the fibroblast subtypes. The color scale represents the scaled expression of each gene. The size of the dot represents the percentage of cells expressing the gene of interest.

c. Spatial plots showing the deconvolution score for the other five fibroblast subtypes in the fibroblast-rich spots.

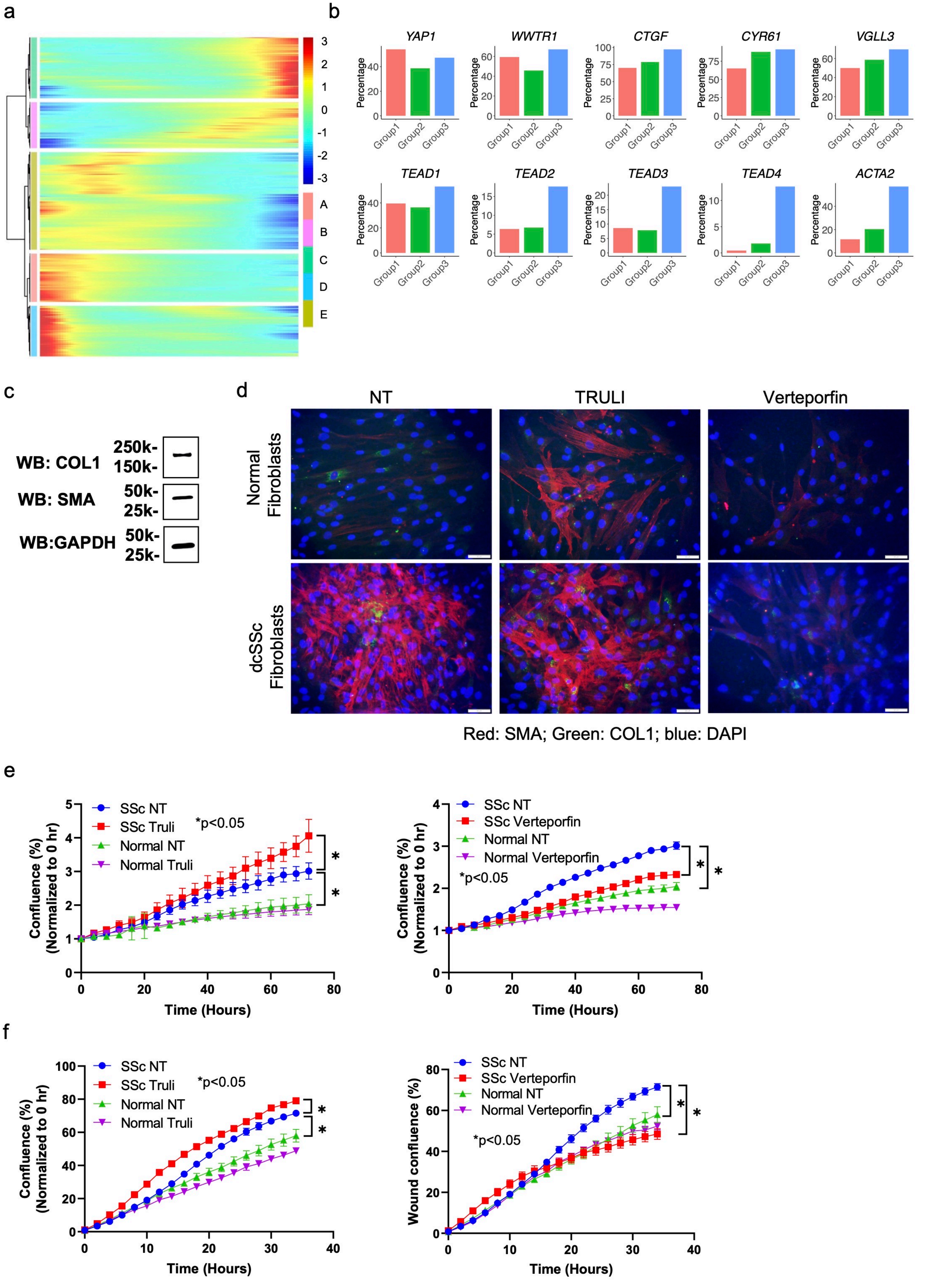
a
A
B
C
D
E
b
YAP1
WWTR1
CTGF
CYR61
VGLL3
TEAD1
TEAD2
TEAD3
TEAD4
ACTA2
Percentage
Group1
Group2
Group3
c
WB: COL1
WB: SMA
WB:GAPDH
250k-
150k-
50k-
25k-
d
NT
TRULI
Verteporfin
Normal Fibroblasts
dcSSc Fibroblasts
Red: SMA; Green: COL1; blue: DAPI
e
SSc NT
SSc Truli
Normal NT
Normal Truli
SSc Verteporfin
Normal Verteporfin
*p<0.05
Confluence (%) (Normalized to 0 hr)
Time (Hours)
f
Wound confluence (%)

Supplementary Fig. 3. Hippo pathways regulates myofibroblast differentiation in SSc skin.

a. Heatmap showing expression of significant marker genes corresponding to five expression patterns that span the fibroblast pseudotime trajectory. Color scale, scaled gene expression across pseudotime.

b. Bar plots showing the percentage of cells expressing the gene in the three fibroblast groups.

c. The basal levels of COL1 and SMA in healthy dermal fibroblasts.

d. Immunofluorescence showing TRULI enhanced while verteporfin Inhibited COL1 and SMA expression in dcSSc fibroblasts. Similar results were observed in normal dermal fibroblasts. However, TRULI appeared to have a smaller effect in these cells than dcSSc fibroblasts. Scale bar = 50 μm.

e. TRULI enhanced while verteporfin blocked proliferation in dcSSc fibroblasts. In contrast, in healthy fibroblasts, these drugs had minimal effects (Truli 10μM and Verteporfin 0.5 μM). A two-way ANOVA test was applied. $P<0.05$ was designated as statistically significant.

f. TRULI enhanced cell migration while verteporfin blocked migration in dcSSc fibroblasts. In healthy fibroblasts, these drugs had minimal effects (Truli 10μM and Verteporfin 0.25 μM). A two-way ANOVA test was applied. $P<0.05$ was designated as statistically significant.

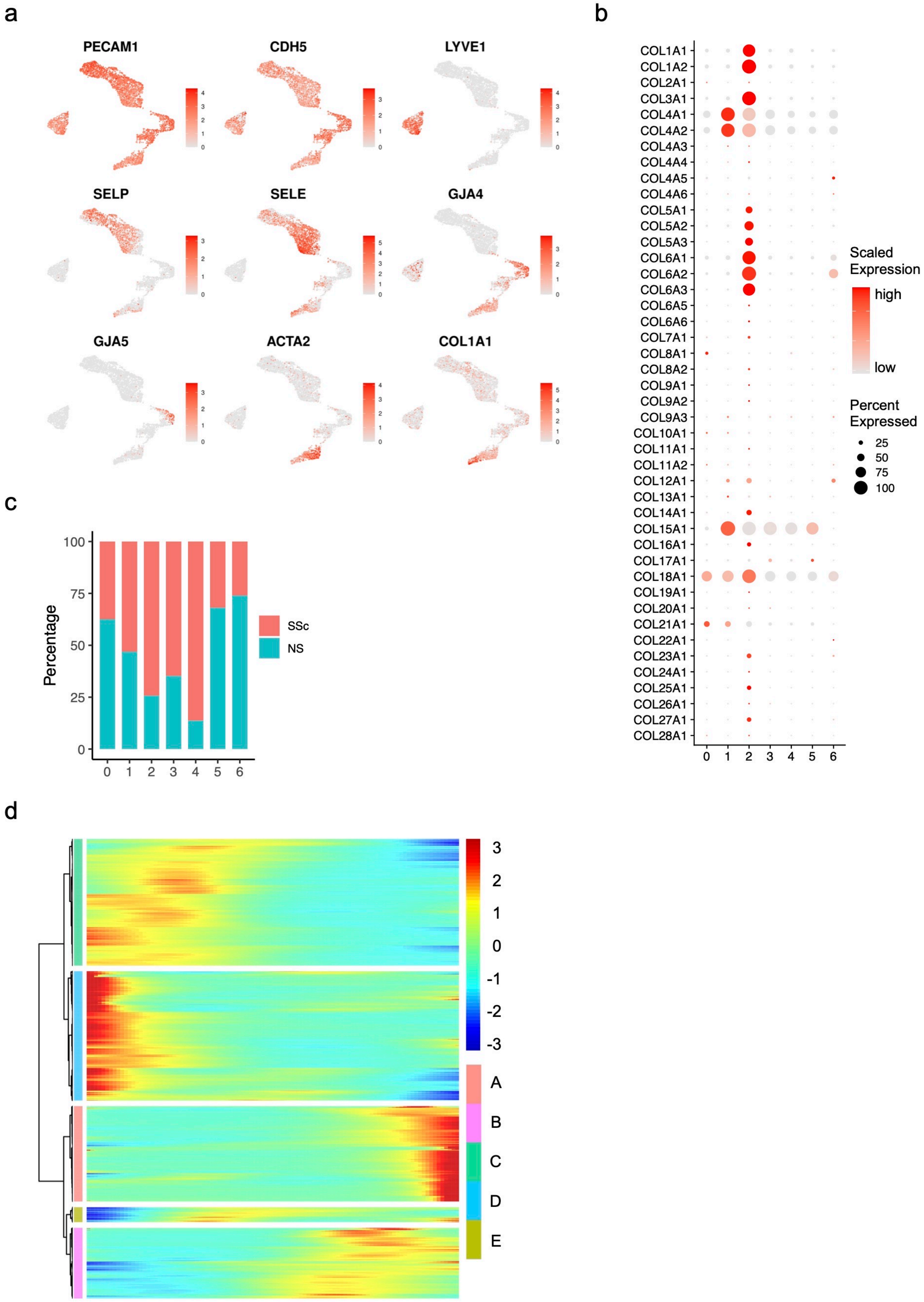
a
PECAM1
CDH5
LYVE1
SELP
SELE
GJA4
GJA5
ACTA2
COL1A1
b
COL1A1
COL1A2
COL2A1
COL3A1
COL4A1
COL4A2
COL4A3
COL4A4
COL4A5
COL4A6
COL5A1
COL5A2
COL5A3
COL6A1
COL6A2
COL6A3
COL6A5
COL6A6
COL7A1
COL8A1
COL8A2
COL9A1
COL9A2
COL9A3
COL10A1
COL11A1
COL11A2
COL12A1
COL13A1
COL14A1
COL15A1
COL16A1
COL17A1
COL18A1
COL19A1
COL20A1
COL21A1
COL22A1
COL23A1
COL24A1
COL25A1
COL26A1
COL27A1
COL28A1
0 1 2 3 4 5 6
Scaled Expression
high
low
Percent Expressed
25
50
75
100
c
Percentage
100
75
50
25
0
0 1 2 3 4 5 6
SSc
NS
d
3
2
1
0
-1
-2
-3
A
B
C
D
E

Supplementary Fig. 4. Characterization of endothelial to mesenchymal transition in SSc skin.

a. UMAP plots showing the expression level of endothelial marker genes and mesenchymal marker genes in the endothelial sub-clusters.

b. Dot plot showing the expression of all the collagen genes across the endothelial sub-clusters. The color scale represents the scaled expression of each gene. The size of the dot represents the percentage of cells expressing the gene of interest.

c. Bar plot showing the abundance composition across the disease conditions for each endothelial sub-cluster.

d. Heatmap showing expression of significant marker genes corresponding to five expression patterns that span the endothelial pseudotime trajectory. Color scale, scaled marker gene expression across pseudotime.

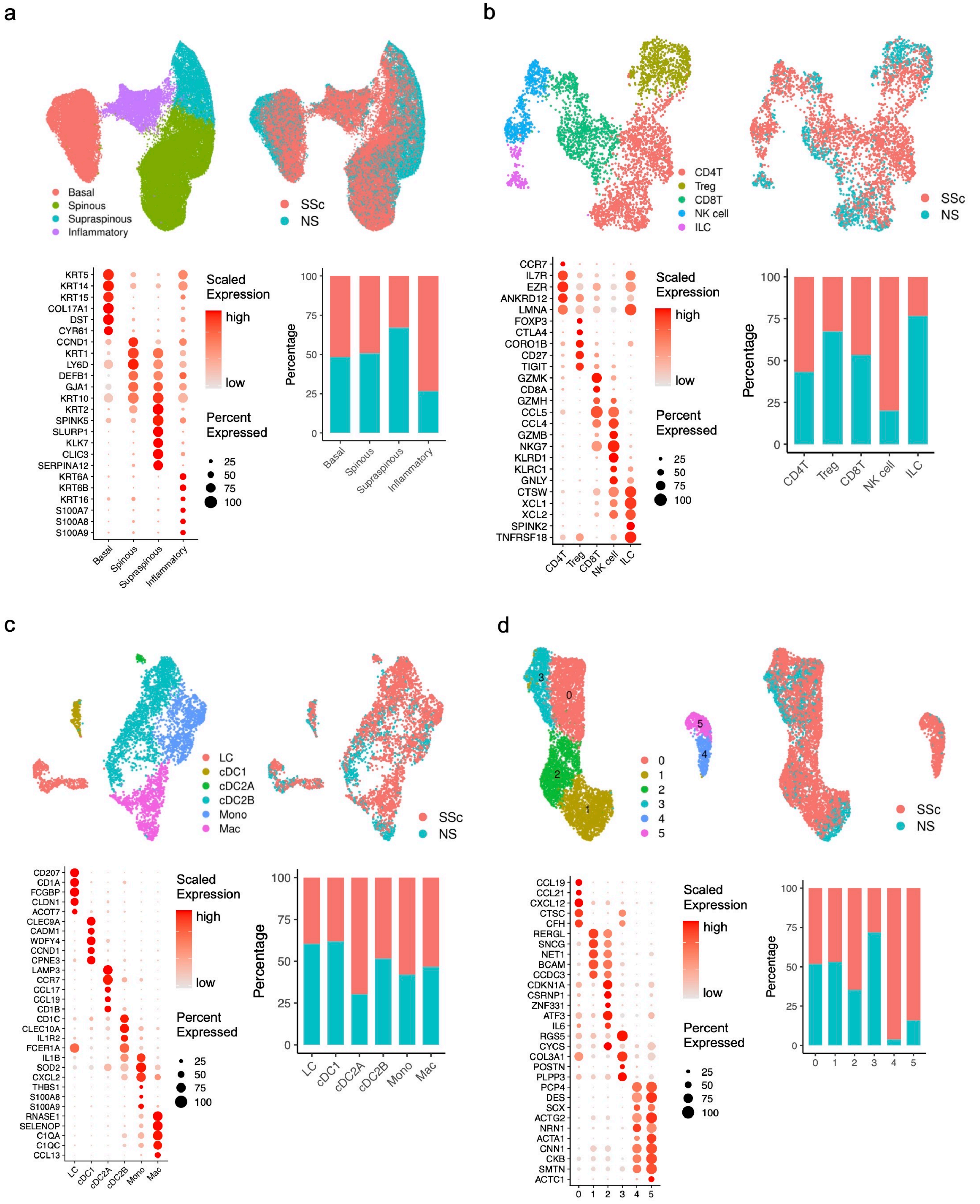

a
Basal
Spinous
Supraspinous
Inflammatory
SSc
NS
KRT5
KRT14
KRT15
COL17A1
DST
CYR61
CCND1
KRT1
LY6D
DEFB1
GJA1
KRT10
KRT2
SPINK5
SLURP1
KLK7
CLIC3
SERPINA12
KRT6A
KRT6B
KRT16
S100A7
S100A8
S100A9
Scaled Expression
high
low
Percent Expressed
25
50
75
100
Percentage
b
CD4T
Treg
CD8T
NK cell
ILC
CCR7
IL7R
EZR
ANKRD12
LMNA
FOXP3
CTLA4
CORO1B
CD27
TIGIT
GZMK
CD8A
GZMH
CCL5
CCL4
GZMB
NKG7
KLRD1
KLRC1
GNLY
CTSW
XCL1
XCL2
SPINK2
TNFRSF18
c
LC
cDC1
cDC2A
cDC2B
Mono
Mac
CD207
CD1A
FCGBP
CLDN1
ACOT7
CLEC9A
CADM1
WDFY4
CCND1
CPNE3
LAMP3
CCR7
CCL17
CCL19
CD1B
CD1C
CLEC10A
IL1R2
FCER1A
IL1B
SOD2
CXCL2
THBS1
S100A8
S100A9
RNASE1
SELENOP
C1QA
C1QC
CCL13
d
0
1
2
3
4
5
CCL19
CCL21
CXCL12
CTSC
CFH
RERGL
SNCG
NET1
BCAM
CCDC3
CDKN1A
CSRNP1
ZNF331
ATF3
IL6
RGS5
CYCS
COL3A1
POSTN
PLPP3
PCP4
DES
SCX
ACTG2
NRN1
ACTA1
CNN1
CKB
SMTN
ACTC1

Supplementary Fig. 5. Identification of keratinocyte, pericyte, smooth muscle, myeloid and T cell subtypes.

a. Identification of keratinocyte subtypes.

b. Identification of T cell subtypes.

c. Identification of myeloid subtypes.

d. Identification of pericyte and smooth muscle subtypes.

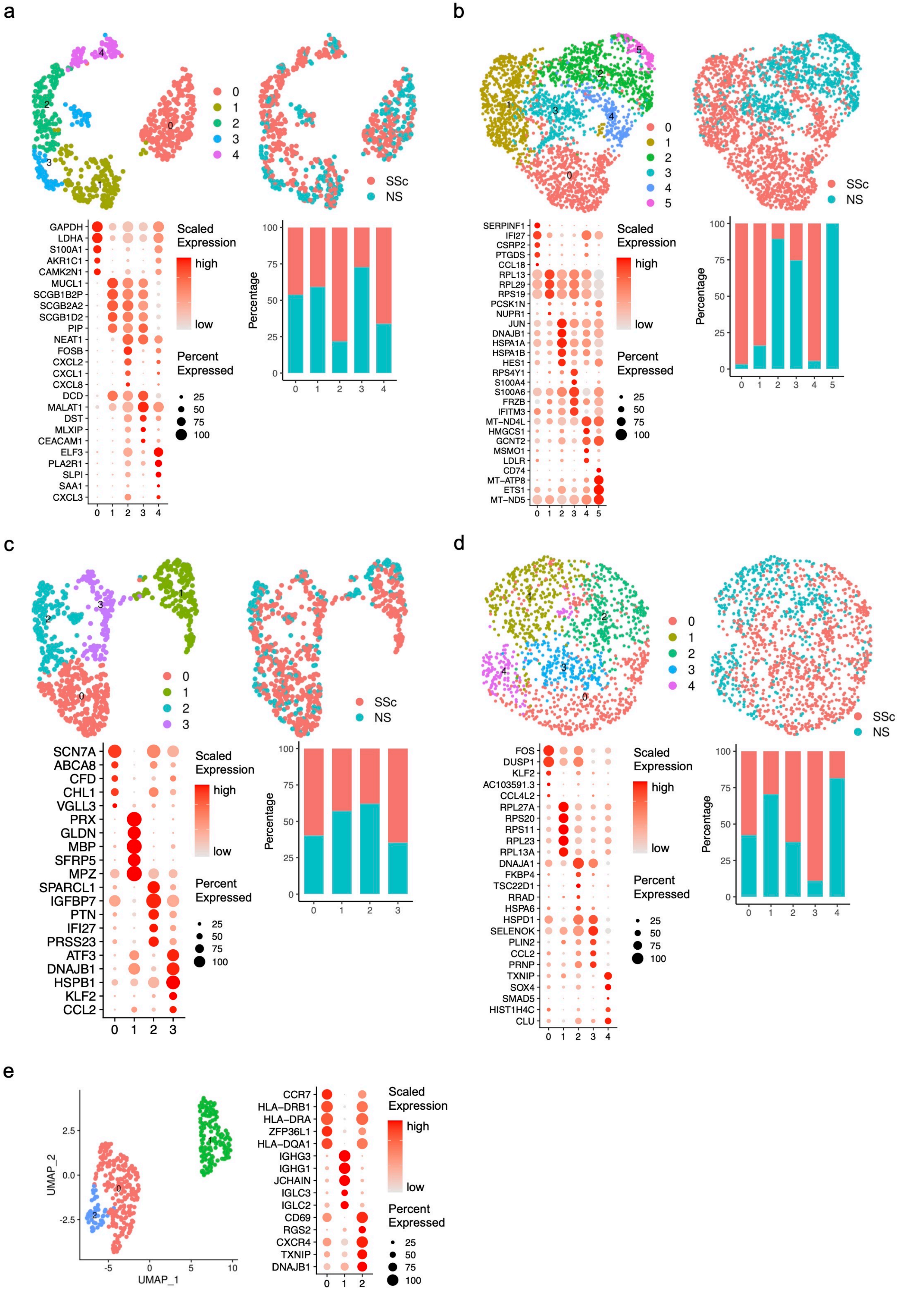

a
0
1
2
3
4
SSc
NS
GAPDH
LDHA
S100A1
AKR1C1
CAMK2N1
MUCL1
SCGB1B2P
SCGB2A2
SCGB1D2
PIP
NEAT1
FOSB
CXCL2
CXCL1
CXCL8
DCD
MALAT1
DST
MLXIP
CEACAM1
ELF3
PLA2R1
SLPI
SAA1
CXCL3
Scaled Expression
high
low
Percent Expressed
25
50
75
100
Percentage
b
5
SERPINF1
IFI27
CSRP2
PTGDS
CCL18
RPL13
RPL29
RPS19
PCSK1N
NUPR1
JUN
DNAJB1
HSPA1A
HSPA1B
HES1
RPS4Y1
S100A4
S100A6
FRZB
IFITM3
MT−ND4L
HMGCS1
GCNT2
MSMO1
LDLR
CD74
MT−ATP8
ETS1
MT−ND5
c
SCN7A
ABCA8
CFD
CHL1
VGLL3
PRX
GLDN
MBP
SFRP5
MPZ
SPARCL1
IGFBP7
PTN
IFI27
PRSS23
ATF3
DNAJB1
HSPB1
KLF2
CCL2
d
FOS
DUSP1
KLF2
AC103591.3
CCL4L2
RPL27A
RPS20
RPS11
RPL23
RPL13A
DNAJA1
FKBP4
TSC22D1
RRAD
HSPA6
HSPD1
SELENOK
PLIN2
CCL2
PRNP
TXNIP
SOX4
SMAD5
HIST1H4C
CLU
e
UMAP_2
UMAP_1
2.5
0.0
−2.5
−5
0
5
10
CCR7
HLA−DRB1
HLA−DRA
ZFP36L1
HLA−DQA1
IGHG3
IGHG1
JCHAIN
IGLC3
IGLC2
CD69
RGS2
CXCR4
TXNIP
DNAJB1

Supplementary Fig. 6. Identification of eccrine gland, melanocyte, nerve, mast cell and B cell subtypes.

a. Identification of eccrine gland subtypes.

b. Identification of melanocyte subtypes.

c. Identification of nerve cell subtypes.

d. Identification of mast cell subtypes.

e. Identification of B cell subtypes.

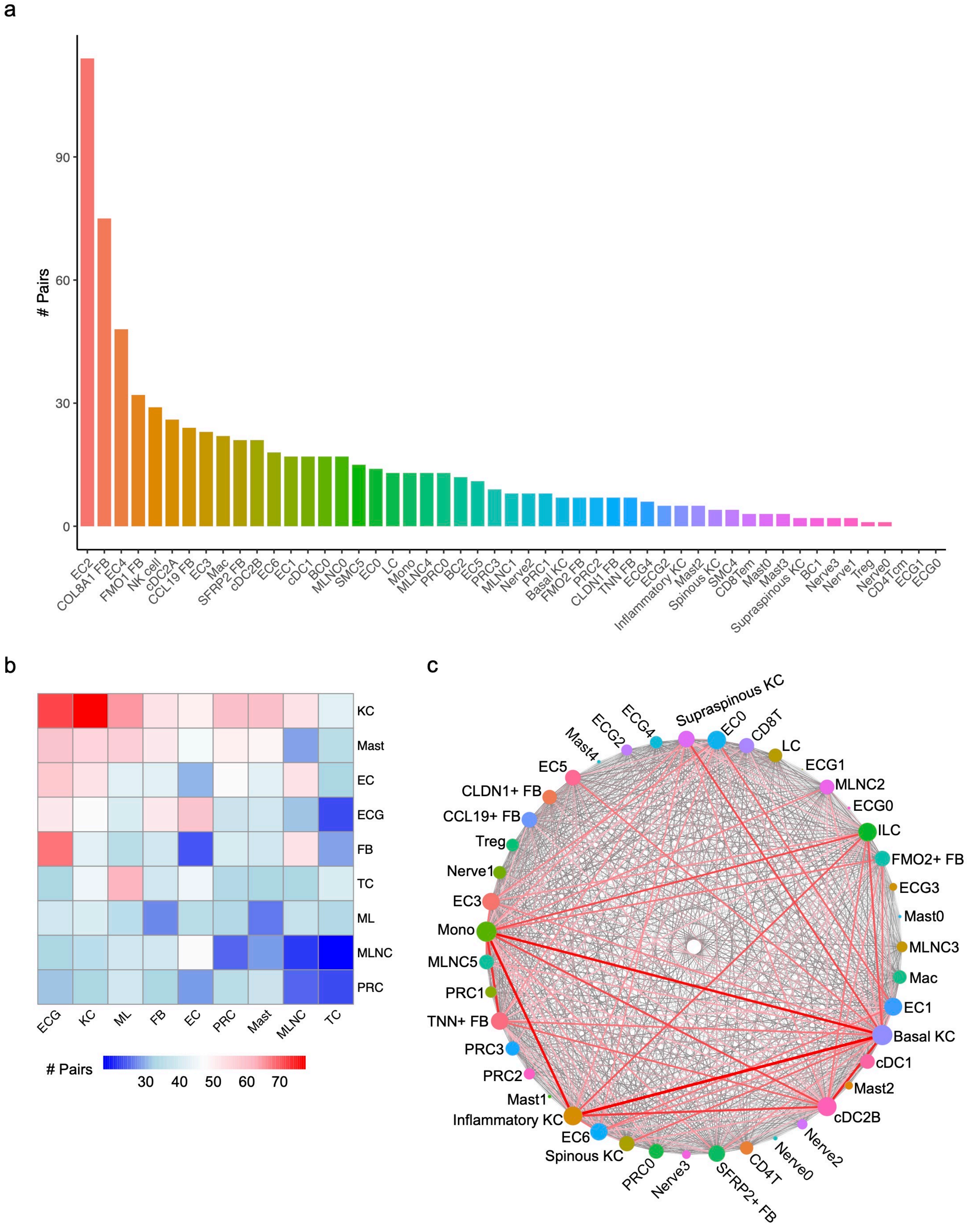
a
Pairs
0
30
60
90
EC2
COL8A1 FB
EC4
FMO1 FB
NK cell
cDC2A
CCL19 FB
EC3
Mac
SFRP2 FB
cDC2B
EC6
EC1
cDC1
BC0
MLNC0
SMC5
EC0
LC
Mono
MLNC4
PRC0
BC2
EC5
PRC3
MLNC1
Nerve2
PRC1
Basal KC
FMO2 FB
PRC2
CLDN1 FB
TNN FB
ECG4
ECG2
Inflammatory KC
Mast2
Spinous KC
SMC4
CD8Tem
Mast0
Mast3
Supraspinous KC
BC1
Nerve3
Nerve1
Treg
Nerve0
CD4Tcm
ECG1
ECG0
b
KC
Mast
EC
ECG
FB
TC
ML
MLNC
PRC
ECG
KC
ML
FB
EC
PRC
Mast
MLNC
TC
Pairs
30
40
50
60
70
c
Supraspinous KC
EC0
CD8T
LC
ECG1
MLNC2
ECG0
ILC
FMO2+ FB
ECG3
Mast0
MLNC3
Mac
EC1
Basal KC
cDC1
Mast2
cDC2B
Nerve2
Nerve0
CD4T
SFRP2+ FB
Nerve3
PRC0
Spinous KC
EC6
Inflammatory KC
Mast1
PRC2
PRC3
TNN+ FB
PRC1
MLNC5
Mono
EC3
Nerve1
Treg
CCL19+ FB
CLDN1+ FB
EC5
Mast4
ECG2
ECG4

Supplementary Fig. 7. Cell-cell communications in the NS samples.

a. Bar plot showing the number of self interactions that are higher in SSc compared to NS in each cell type.

b. Heatmap showing the number of ligand-receptor pairs with interaction scores higher in NS compared to SSc. Row, cell type expressing the ligand; column, cell type expressing the receptor. Color scale, number of ligand-receptor pairs. EC, endothelial cell; FB, fibroblast; ML, myeloid cell; Mast, mast cell; KC, keratinocyte; PRC, pericyte. TC, T cell; ECG, eccrine gland cell; MLNC, melanocyte.

c. Connectome web analysis of interacting subtypes in the NS samples. Vertex (colored cell node) size is proportional to the number of interactions to and from that cell type, whereas the thickness of the connecting lines is proportional to the number of interactions between two nodes. The Vertex size and the line thickness follow the same scales as in Fig. 6b.

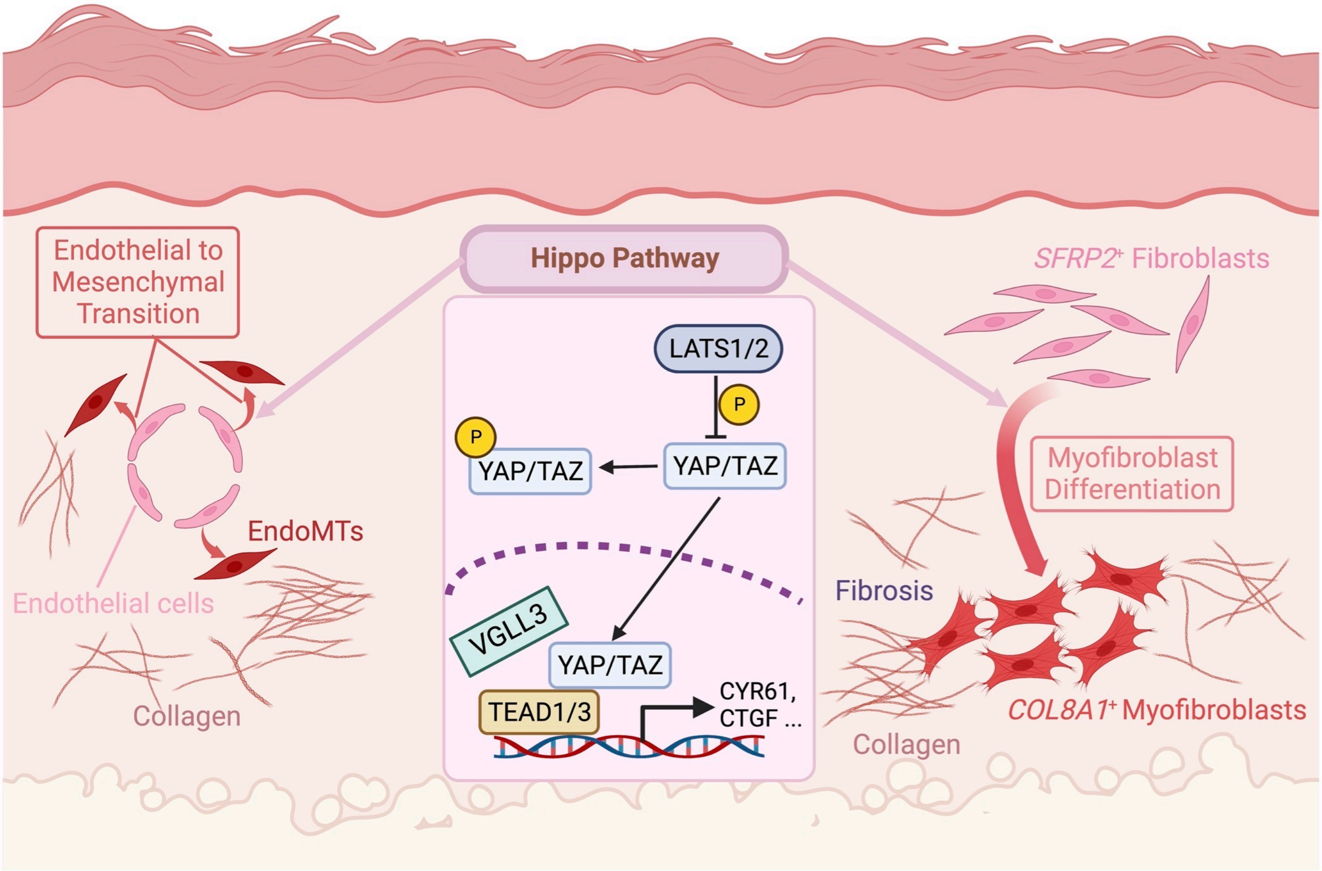

Supplementary Fig. 8. Schematic overview of the critical role of Hippo pathway in modulating myofibroblast differentiation and endothelial to mesenchymal transition in SSc skin. Created with BioRender.com.
