# Supplementary material for: Systems-based identification of the Hippo pathway for promoting fibrotic mesenchymal differentiation in systemic sclerosis
Source: Nat Commun. 2024 Jan 3;15:210. doi: 10.1038/s41467-023-44645-6 (PMC10764940; doi:10.1038/s41467-023-44645-6)
Supplement: Supplementary file 3 — Description of Additional Supplementary Files [file 41467_2023_44645_MOESM3_ESM.pdf]

Description of Additional Supplementary Files

Supplementary Data 1. Demographics for systemic sclerosis patients.

Supplementary Data 2. Cell type, subtype, and sub-cluster marker genes in the scRNA-seq analyses.

Supplementary Data 3. Genes induced in fibroblasts after stimulation with TGF-β or IL-4 from bulk RNA-seq experiments.

Supplementary Data 4. Genes in the five expression patterns along the fibroblast or endothelial pseudotime in the scRNA-seq analyses.

Supplementary Data 5. Common up-regulated genes in fibroblast group 3 vs group 1, 2 and endothelial sub-cluster 2 vs sub-cluster 0, 1.

Supplementary Data 6. Ligand-receptor pairs specific to SSc fibroblast and endothelial subtypes plotted in Fig. 6c.

Supplementary Data 7. Antibodies used in this study.
