# Supplementary material for: Systems-based identification of the Hippo pathway for promoting fibrotic mesenchymal differentiation in systemic sclerosis
Source: Nat Commun. 2024 Jan 3;15:210. doi: 10.1038/s41467-023-44645-6 (PMC10764940; doi:10.1038/s41467-023-44645-6)
Supplement: Supplementary file 5 — Reporting Summary [file 41467_2023_44645_MOESM5_ESM.pdf]

nature portfolio

Corresponding author(s): Johann E. Gudjosson

Last updated by author(s): Dec 5, 2023

Reporting Summary

Nature Portfolio wishes to improve the reproducibility of the work that we publish. This form provides structure for consistency and transparency in reporting. For further information on Nature Portfolio policies, see our Editorial Policies and the Editorial Policy Checklist.

Statistics

For all statistical analyses, confirm that the following items are present in the figure legend, table legend, main text, or Methods section.

n/a	Confirmed	
☐	☒	The exact sample size (*n*) for each experimental group/condition, given as a discrete number and unit of measurement
☐	☒	A statement on whether measurements were taken from distinct samples or whether the same sample was measured repeatedly
☐	☒	The statistical test(s) used AND whether they are one- or two-sided<br>*Only common tests should be described solely by name; describe more complex techniques in the Methods section.*
☐	☒	A description of all covariates tested
☐	☒	A description of any assumptions or corrections, such as tests of normality and adjustment for multiple comparisons
☐	☒	A full description of the statistical parameters including central tendency (e.g. means) or other basic estimates (e.g. regression coefficient) AND variation (e.g. standard deviation) or associated estimates of uncertainty (e.g. confidence intervals)
☐	☒	For null hypothesis testing, the test statistic (e.g. *F*, *t*, *r*) with confidence intervals, effect sizes, degrees of freedom and *P* value noted<br>*Give P values as exact values whenever suitable.*
☐	☒	For Bayesian analysis, information on the choice of priors and Markov chain Monte Carlo settings
☐	☒	For hierarchical and complex designs, identification of the appropriate level for tests and full reporting of outcomes
☐	☒	Estimates of effect sizes (e.g. Cohen's *d*, Pearson's *r*), indicating how they were calculated

Our web collection on statistics for biologists contains articles on many of the points above.

Software and code

Policy information about availability of computer code

Data collection	No software was used for single cell or spatial-seq data acquisition.
Data analysis	10X Genomics software cellranger (version 3.1.0), spaceranger (version 1.3.0), R package Seurat (version 3.1.2), monocle (version 2.10.1), and python package CellphoneDB (version 2.0.0) were used to analyze the scRNA-seq and spatial-seq data.

For manuscripts utilizing custom algorithms or software that are central to the research but not yet described in published literature, software must be made available to editors and reviewers. We strongly encourage code deposition in a community repository (e.g. GitHub). See the Nature Portfolio guidelines for submitting code & software for further information.

Data

Policy information about availability of data

All manuscripts must include a data availability statement. This statement should provide the following information, where applicable:

- Accession codes, unique identifiers, or web links for publicly available datasets
- A description of any restrictions on data availability
- For clinical datasets or third party data, please ensure that the statement adheres to our policy

The single cell RNA-seq and spatial-seq data generated in this study have been deposited in the GEO database under accession code GSE249279.

Research involving human participants, their data, or biological material

Policy information about studies with human participants or human data. See also policy information about sex, gender (identity/presentation), and sexual orientation and race, ethnicity and racism.

Reporting on sex and gender	The sex information is reported in supplemental table 1. Sex or gender was not considered in the study design.
Reporting on race, ethnicity, or other socially relevant groupings	Race and Ethnicity information is reported in supplemental table 1. These were not considered in the study design.
Population characteristics	Skin biopsy specimens were obtained from patients with psoriasis at University of Michigan-Ann Arbor. The population characteristics, including age, are reported in supplemental table 1.
Recruitment	Patients with psoriasis are seen at the hospital of University of Michigan-Ann Arbor. Patients were off systemic treatment and off any topical agents for at least 2 weeks prior to study time. All patients were asked if they wish to participate in the ongoing research studies, and all of them consented.
Ethics oversight	Informed written consent was obtained from human subjects under a protocol approved by the institutional review boards of University of Michigan-Ann Arbor. This study was conducted according to the Declaration of Helsinki Principles.

Note that full information on the approval of the study protocol must also be provided in the manuscript.

Field-specific reporting

Please select the one below that is the best fit for your research. If you are not sure, read the appropriate sections before making your selection.

☒ Life sciences ☐ Behavioural & social sciences ☐ Ecological, evolutionary & environmental sciences

For a reference copy of the document with all sections, see nature.com/documents/nr-reporting-summary-flat.pdf

Life sciences study design

All studies must disclose on these points even when the disclosure is negative.

Sample size	22 systemic sclerosis patients and 18 healthy donors were recruited for single cell RNA sequencing, and additional 4 systemic sclerosis patients were recruited for spatial sequencing. No sample size calculation was performed, the sample sizes were chosen based on the number of patient or healthy donors we could recruit.
Data exclusions	Cells with less than 500 transcripts or 100 genes, or more than 15% of mitochondrial expression were filtered out as low-quality cells in scRNA-seq data analysis. After initial clustering of the remaining cells, clusters showing low transcript number and high mitochondrial expression were excluded to avoid the analysis of dead cells or empty droplets with fragments.
Replication	The samples in the same disease condition can be considered biological replicates. The replications were successful as the biological replicates showed similar gene expression profiles. There are in total 18 healthy and 22 systemic sclerosis samples in scRNA-seq. There are 4 systemic sclerosis samples in spatial-seq.
Randomization	Not applicable, as no comparison of experimental groups is performed. All comparisons presented are performed on cell groups from different disease conditions or different cell types.
Blinding	Not applicable. In the scRNA-seq analysis, the goal is to analyze gene expression at the level of individual cells. Each cell serves as a data point, and blinding to the identity or condition of individual cells within the dataset is not practical.

Reporting for specific materials, systems and methods

We require information from authors about some types of materials, experimental systems and methods used in many studies. Here, indicate whether each material, system or method listed is relevant to your study. If you are not sure if a list item applies to your research, read the appropriate section before selecting a response.

Materials & experimental systems

n/a	Involved in the study	
☐	☒	Antibodies
☒	☐	Eukaryotic cell lines
☒	☐	Palaeontology and archaeology
☒	☐	Animals and other organisms
☒	☐	Clinical data
☒	☐	Dual use research of concern
☒	☐	Plants

Methods

n/a	Involved in the study	
☒	☐	ChIP-seq
☒	☐	Flow cytometry
☒	☐	MRI-based neuroimaging

Antibodies

Antibodies used

. Primary rabbit antibodies were used: anti-alpha smooth muscle actin (αSMA), anti-Col1A1, anti-Vgll3, anti- Vimentin, anti-YAP anti-TEAD1, anti-CD31 antibodies from (Abcam, cat # ab5694, LSB, cat # LS-C343921, Abcam, cat # ab254938, Abcam, cat # ab 92542, Cell Signaling, cat # 12292 and Abcam, cat # ab 32457, respectively). Along with rabbit primary antibodies, appropriate mouse-antihuman antibodies were also used: anti-αSMA (cat # ab 254938), anti- Vimentin (cat # ab 8978), anti-CD31(cat # ab 9498), all from Abcam and TEAD3 antibody from (Abnova, cat # H00007005). All primary rabbit antibodies mentioned above were diluted 1:100, except CD31 1:250 dilution in blocking solution and coincubated with appropriate primary mouse antibodies (α-SMA, Vimentin and TEAD3 1:50 dilutions and CD31 1:100 overnight at 4°C. Appropriate negative (no primary or secondary antibodies or isotype control antibodies: rabbit IgG (ab172730), mouse IgG1 (ab 280974) both from Abcam, IgG2ak (14-4724-82) from Invitrogen, antibodies were stained in parallel with each set of the slides mentioned above. Slides were then washed three times for 5 min each with phosphate-buffered saline/ Tween 20 (PBST). All slides were then incubated with secondary antibodies fluorochrome-conjugated Alexa Fluor 594 conjugated anti-rabbit IgG (711-585-152) and Alexa Fluor 488 conjugated anti-mouse IgG (715-545-151) from Jackson Immuno Research.

Validation

Antibodies validated by the manufacturer were used. Antibodies were initially characterized and titrated by staining an appropriate positive control at several dilutions and a negative control.

Plants

Seed stocks

Report on the source of all seed stocks or other plant material used. If applicable, state the seed stock centre and catalogue number. If plant specimens were collected from the field, describe the collection location, date and sampling procedures.

Novel plant genotypes

Describe the methods by which all novel plant genotypes were produced. This includes those generated by transgenic approaches, gene editing, chemical/radiation-based mutagenesis and hybridization. For transgenic lines, describe the transformation method, the number of independent lines analyzed and the generation upon which experiments were performed. For gene-edited lines, describe the editor used, the endogenous sequence targeted for editing, the targeting guide RNA sequence (if applicable) and how the editor was applied.

Authentication

Describe any authentication procedures for each seed stock used or novel genotype generated. Describe any experiments used to assess the effect of a mutation and, where applicable, how potential secondary effects (e.g. second site T-DNA insertions, mosiacism, off-target gene editing) were examined.
