# Supplementary material for: Systems-based identification of the Hippo pathway for promoting fibrotic mesenchymal differentiation in systemic sclerosis
Source: Nat Commun. 2024 Jan 3;15:210. doi: 10.1038/s41467-023-44645-6 (PMC10764940; doi:10.1038/s41467-023-44645-6)
Supplement: Supplementary file 6 — Source Data [file 41467_2023_44645_MOESM6_ESM.zip › Source_Data/Source_Data_Legends.docx]

**Source Data**

**Source Data 1. Original western blots, related to Fig. 3k.**

**Source Data 2. Underlying data for Fig. 3.**

**Source Data 3. Original western blots, related to Fig. 5b.**

**Source Data 4. Underlying data for Fig. 5.**
